# News and Coming Events

**Published:** 1908-02-15

**Authors:** 


### NEWS AND COMING EVENTS.

Dr. Kenneth R. Hay has been appointed medical officer to the Royal Institution of Great Britain.

Mrs. Mary Ramsey Wood, a native of East Tennessee, has died in Bristol, Va., at what is claimed to be the well-authenticated age of 120 years.

The annual general meeting of the Cancer Hospital (Free), Fulham Road, S.W., will be held on Wednesday, February 26, 1908, at 4 p.m. precisely.

The 58th annual general meeting of the London Homœopathic Hospital will take place in the board-room of the hospital on Friday, February 28, at 3.30 p.m., the Right Hon. the Earl Cawdor presiding.

Mr. Charters J. Symonds, M.S., F.R.C.S., surgeon to Guy's Hospital, who is delivering the Lettsomian Lectures before the Medical Society of London, will discuss certain special forms of abdominal tuberculosis and cancer.

After an interval of ten years, the *New York Polyclinic Journal*, a monthly devoted to the discussion of matters of interest to the general practitioner, will resume publication. Dr. Charles H. Chetwood will be the new editor.

Dr. B. A. Peters, one of the house surgeons at the Royal Southern Hospital, Liverpool, has been awarded the bronze medal of the Royal Humane Society for his gallant rescue of a young lady off the coast of Anglesey last autumn.

St. Mary's Hospital has lately established a special out-patient department devoted to diseases of the nervous system. Dr. Wilfred Harris, senior physician to out-patients, has been appointed physician-in-charge of the new department.

Since 1902 3,395 papers on cancer have been published. Of these 1,960 are in German, 607 in English, 549 in French, 113 in Italian, 70 in Russian and Polish, 37 in Norwegian and Swedish, 22 in Spanish, 16 in Dutch, 9 in Hungarian, 7 in Portuguese, 3 in Japanese, and 2 in Greek.

His Majesty the King has sent ten brace of pheasants and his annual subscription of ten guineas to the Royal Waterloo Hospital. This institution is the oldest one in the Empire for the treatment of children, and was founded in 1816 by the late Queen Victoria's father, H.R.H. the Duke of Kent.

After the annual meeting of the After Care Association for Poor Persons Discharged Recovered from Asylums for the Insane held at 48 Wimpole Street, a paper on "Convalescence from Mental Disorders and its Difficulties" was read by Dr. G. H. Savage, the Treasurer, who drew attention to the necessity for convalescent institutions for patients discharged from asylums.

The Queen has accepted a copy of the "King's College Hospital Book of Cooking Recipes"—a collection of 600 old family recipes contributed by friends of the hospital. It is published at 1s. net, and the proceeds are devoted to the Hospital Removal Fund. Building in connection with the new hospital to be erected at Denmark Hill will be begun almost at once. The first contract embraces the whole of the out-patient, casualty, and bathing departments. Tenders for the erection of this block have been received, and the committee have now accepted that of Messrs. Foster and Dicksee, Limited.

In 1909 the Royal College of Physicians of London will award the Weber-Parkes prize of 150 guineas and a silver medal in memory of the late Dr. E. A. Parkes. The adjudicators have selected as the subject of the essay for that occasion "The value of bacterial products in protecting against or in curing tuberculous diseases, with special reference to pulmonary tuberculosis in man."

Owing to the drastic measures adopted by the Sheffield Corporation the purity of the Sheffield atmosphere has been much improved. Last year Sheffield enjoyed 1,437 hours of sunshine, or 368 more than Manchester, 367 more than Bradford, 265 more than Glasgow, 189 more than Leeds, 186 more than Birmingham, 158 more than Edinburgh, and 53 more than York.

In connection with the Congress of the Royal Institute of Public Health at Buxton, from July 18 to July 24 next, under the presidency of the Right Hon. Victor Cavendish, M.P., a special Exhibition will be held in the large Buxton Pavilion. No Exhibition has been held in connection with the Institute since the meeting in Blackpool seven years ago, and the present, therefore, offers an exceptional opportunity for the manufacturers of articles that are specially connected with the Public Health services to bring them before possible customers.

The weekly statement of the number of persons in receipt of Poor-law relief, issued by the Local Government Board, shows that on January 18 there were 129,590 paupers in London, of whom 81,179 were in the workhouses and 48,411 on the outdoor relief lists. The total was higher by 5,612 than that for the corresponding period of 1907. The rate of pauperism per thousand of the population was 27.2, as against 26.3 in January 1907. The figures for 1906 were: 127,107 paupers, or 27.1 per 1,000 of the population; and for 1905, 128,996 paupers, or 27.7 per 1,000 of the population.

Sir John Denis Macdonald, K.C.B., R.N., F.R.S., M.D., retired Inspector-General of Hospitals and Fleets, died at Southall on February 7 at the age of 81. Sir John Macdonald was educated at Cork School of Medicine and King's College Medical School, and entered the Navy as assistant surgeon in 1849. He did valuable scientific work on the exploring and surveying expedition of H.M.S. *Herald*, and was elected F.R.S. in 1859. For nine years he was Professor of Naval Hygiene at Netley. He published numerous scientific papers, and was the recipient of several medals in recognition of his important contributions to microscopical science.

Surgeon-General William Henry Rean, M.D., died on Feb. 6 at the age of 81. During his student days he was one of the first pupils of Sir Isaac Pitman, and he used the then new system of shorthand when attending his hospital lectures. He was house surgeon at the Middlesex Hospital, and entered the East India Company's service in 1853. In 1857 he served with the Madras Fusiliers in the attempted relief of Cawnpore, and was medical officer on board the *Bramaputra*, which was despatched up-stream from Allahabad, and arrived at Cawnpore shortly after the massacre. At Cawnpore he became chief medical officer in charge of the standing hospital, but unluckily contracted cholera, and was despatched with the first batch of invalids to Calcutta by river steamers. After the Mutiny he served as senior medical officer in the Andaman Islands for nine years. Before he retired, in 1881, after 33 years' service in India, he was deputy surgeon-general at Kampti.

